# A New Inflammation-Related Risk Model for Predicting Hepatocellular Carcinoma Prognosis

**DOI:** 10.1155/2022/5396128

**Published:** 2022-05-05

**Authors:** Mindan Xing, Jia Li

**Affiliations:** ^1^Nankai University School of Medicine, No. 94 Weijin Road, Nankai District, Tianjin 300071, China; ^2^Tianjin Second People's Hospital, No. 7, Sudi South Road, Nankai District, Tianjin 300192, China

## Abstract

**Background:**

Hepatocellular carcinoma (HCC) is characterized by a poor prognosis. Inflammation has a vital role in the formation and development of HCC. However, the prediction of HCC prognosis using inflammation-related genes (IRGs) remains elusive. In this study, we constructed a new IRG risk model to predict the HCC prognosis.

**Results:**

HCC-related RNA expression profiles and their corresponding clinical data were downloaded from TCGA and ICGC databases to explore the IRGs' predicting ability. Seven hundred thirty-seven IRGs from GeneCards were used as candidate genes to construct the model. The associations of overall survival (OS) with IRGs were evaluated using the log-rank test and univariate Cox analysis, and 32 out of 737 IRGs showed predicting the potential for HCC prognosis. These IRGs were further analyzed using the least absolute shrinkage and selection operator (LASSO) and multivariate Cox analyses. Finally, 6 IRGs were included in an IRG risk model. Based on the cut-off of the risk score calculated according to the IRG risk model, HCC samples were divided into the high-risk and the low-risk groups. The OS of patients was lower in the high-risk group than in the low-risk group (*P* < 0.05). The area under the receiver operating characteristic curve (AUC) of the risk score was 0.78 for 3-year survival. Univariate Cox and multivariate Cox analyses revealed that the risk score was an independent risk factor for HCC prognosis. The KEGG and GO enrichment analysis results further showed that the risk scores were closely related to inflammatory and immune pathways. In addition, the ssGSEA demonstrated that several immune cells and some immune-related pathways were negatively correlated with the risk score.

**Conclusions:**

The new IRG risk score was an independent risk factor for HCC prognosis and could be used to assess the immune status of the HCC microenvironment.

## 1. Introduction

Liver cancer is one of the most common malignancies and the fourth leading cause of cancer-related mortality worldwide [1]. Each year, there are approximately 841,000 new cases and 782,000 patient deaths from this disease, and these numbers continue to rise [2]. As the most common form of liver cancer [1, 3], hepatocellular carcinoma (HCC) is the third-largest cause of cancer-related deaths worldwide [4]. Due to the high heterogeneity of HCC, diagnosis and prognosis are challenging.

HCC is a prototypical inflammation-associated cancer [5]. It has been estimated that 90% of HCC occurs from the underlying chronic i4nflammation of the liver, the induction of fibrosis, and subsequent cirrhosis [6]. The liver has a strong self-repair ability. Differentiated liver cells can reenter the cell cycle to repair the cells after acute liver injury [7]. However, the persistence of inflammation, sustained cell death, compensatory proliferation, activation of nonparenchymal cells, and an altered immune response can promote liver fibrosis and, in turn, lead to tumorigenesis [6–8]. Along with the occurrence of tumors, inflammatory signals, cellular stress, epigenetic modifications [9], mitochondrial stress signaling [10], and the immune system may change [11], thus posing significant challenges to the treatment and prognosis evaluation of HCC.

Some studies have demonstrated that some inflammation markers are associated with the prognosis of HCC. The most common biomarkers for HCC are serological parameters, including thrombocytosis, leukocytosis, hypoproteinemia, and plasma fibrinogen [12, 13]. Lin et al. [14] have constructed an inflammation-related risk for prognostic prediction in HCC. However, the AUC of the risk score for 3-year survival was only 0.605. In this study, we constructed a new inflammation-related risk model based on inflammation-related genes (IRGs) from the GeneCards to more accurately predict the HCC prognosis.

## 2. Methods

### 2.1. Data Sets

IRGs were extracted from GeneCards (https://www.genecards.org/), and 737 IRGs with a relevance score > 3 were selected for further analysis. Three hundred seventy-one HCC samples' RNA sequencing data and related clinical information were extracted from TCGA database through the Genomic Data Commons (GDC) tool (https://portal.gdc.cancer.gov). Another 231 HCC samples' RNA sequencing data and clinical information were obtained from the ICGC website (https://dcc.icgc.org/projects/LIRI-JP). To confirm detection reliability, genes with read counts equal to 0 in more than 25% of the samples were removed from further analysis. Patients whose survival time was <0.1 months or with incomplete survival data were removed. Finally, 335 tumor samples from TCGA cohort and 231 tumor samples from the ICGC cohort were enrolled in this study.

### 2.2. Construction of the Inflammation-Related Genes Risk Model

We firstly conducted univariate Cox regression and log-rank test using the *survival* R package to identify IRGs associated with the overall survival (OS) in TCGA cohort. The IRGs with *P* < 0.05 in both two analyses were retained. Next, data were analyzed by the least absolute shrinkage and selection operator (LASSO) regression using the *glmnet* R package. Subsequently, the multivariate Cox regression analysis stepwise method was performed using the *survival* package and *My.stepwise* R package to establish an optimal IRG risk model. The risk score was computed as follows: risk score = ∑_*i*_^6^*Xi* × *Yi* (*X*: coefficients, *Y*: gene expression level). We used the *survminer* R package to determine the optional cut-off value of the risk score. The HCC samples were finally divided into high-risk and low-risk groups based on the cut-off value.

### 2.3. Performance Assessment Inflammation-Related Risk Model

The Kaplan-Meier survival analysis and log-rank test were performed using the *survival* and *survminer* R packages to display and compare the OS of the high-risk and the low-risk groups. Principal component analysis (PCA) was used to examine the difference between the two risk groups. The R package *timeROC* was used to establish a time-dependent receiver operating characteristic curve (ROC) to check the accuracy of the risk score in predicting the outcomes of HCC patients. To explore the prognostic factors of HCC, univariate and multivariate Cox analyses were conducted.

### 2.4. Functional Enrichment Analysis of the DEGs between the Two Risk Groups

We used *DESeq2*, *edgeR*, and *limma* R packages to select differentially expressional genes (DEGs) between high-risk and low-risk groups in TCGA cohort. The screening criteria were ∣log2Fold − Change | >1 and *P* < 0.05. Based on these DEGs, Gene Ontology (GO) and Kyoto Encyclopedia of Genes and Genomes (KEGG) analyses were performed using the *ClusterProfiler* package.

### 2.5. Comparison of the Immune Status between Two Risk Groups

The *ESTIMATE* R package was used to calculate the immune score, stromal score, and ESTIMATE score, reflecting the tumor purity and the characteristics of the tumor microenvironment (TME) [15]. The *gsva* R package was utilized to conduct the single-sample gene set enrichment analysis (ssGSEA) to calculate the scores of infiltrating immune cells and evaluate immune-related pathways' activity in TCGA and ICGC cohorts.

### 2.6. Acquisition of Immunotherapeutic Cohorts and Collection of Clinical Information

To understand whether the risk score could be used to predict the efficacy of immunotherapy, we found the IMvigor210 cohort (http://research-pub.Gene.com/imvigor210corebiologies) after a systematic search of the public databases. The IMvigor210 cohort investigated the effectiveness of anti-PD-L1 antibody (pembrolizumab) in patients with advanced urothelial cancer. The complete transcriptome data and detailed clinical information were enrolled.

### 2.7. Statistical Analysis

One-way ANOVA and Kruskal-Wallis tests were used for multiple comparisons [16]. The Wilcoxon test was used to test the significant difference between the two groups. The Kaplan-Meier survival analysis was used to generate survival curves, and the significance of differences was compared using the log-rank test. Hazard ratios (HRs) and 95% confidence interval (CI) were calculated using univariate Cox and multivariate Cox analyses. Two-sided *P* < 0.05 was considered statistically significant. The R 4.1.1 software was used to perform all data processing.

## 3. Results

### 3.1. Clinical Cohorts

**Figure 1:**
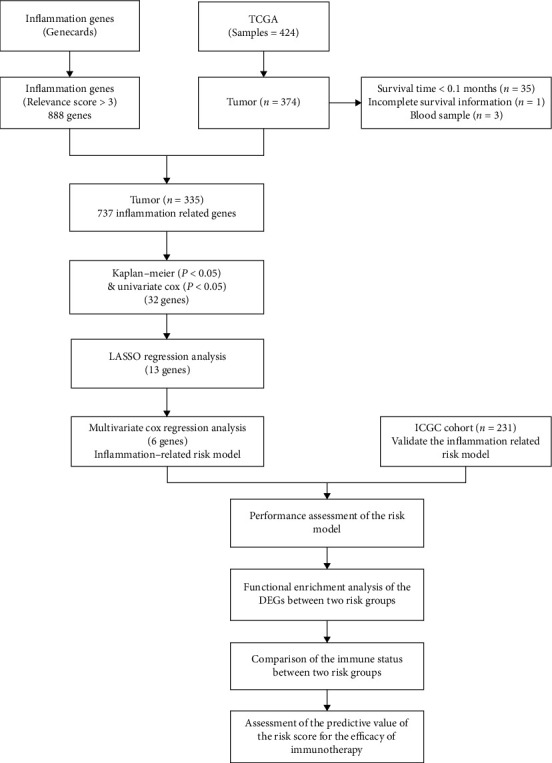
The workflow chart.

The workflow chart of this study is shown in Figure 1. A total of 566 HCC patients and 348 patients with advanced urothelial cancer were included in the analysis. Data of 335 HCC patients from TCGA database, data of 231 HCC patients from the ICGC database, and 348 patients with advanced urothelial cancer from the IMvigor210 cohort were included in the analysis. TCGA cohort was used to construct the IRG risk model, and the ICGC cohort was used for validation. The IMvirgor210 cohort was used to evaluate the predictive value of the risk model for immunotherapy efficacy. The detailed clinical information of HCC patients are shown in Table 1, and the clinical information of patients in the IMvirgor210 cohort are shown in Table 2.

### 3.2. Conduction and Validation of the Inflammation-Related Genes Risk Model

**Figure 2:**
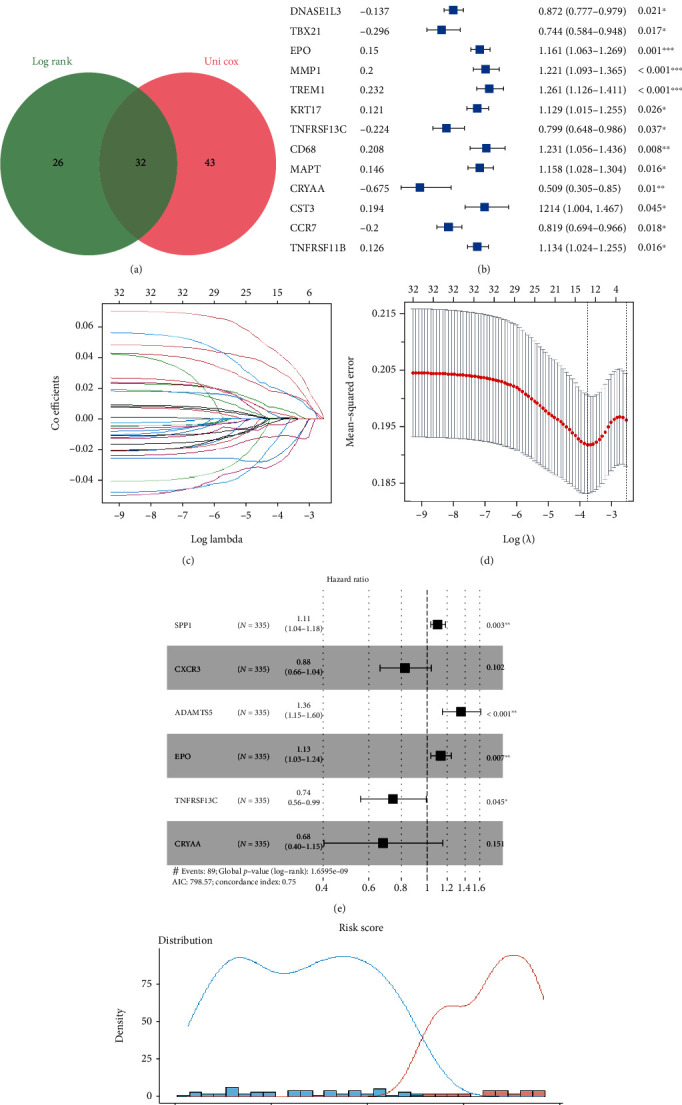
Construction of the inflammation-related genes risk model in TCGA cohort. (a) Venn diagram used to display the 32 IRGs with predicting prognosis ability. (b) Results of the univariate Cox analysis of 32 IRGs. (c) Plots for LASSO expression coefficients of 32 IRGs. (d) Cross-validation for tuning the parameter selection in the LASSO regression. (e) The relationship between 6 IRGs and HCC prognosis, ^∗^*P* < 0.05, ^∗∗^*P* < 0.01, ^∗∗∗^*P* < 0.001. (f) The optimal cut-off point to dichotomize risk score into low-risk and high-risk groups was determined by the *survminer* R package. The optimal cut-off point was 0.23.

Using univariate Cox analysis and log-rank test, 32 out of 737 IRGs showed predicting prognosis ability in HCC (Figures 2(a) and 2(b)). Then, the 32 IRGs were screened by the LASSO regression analysis, and 13 IRGs were obtained for further analysis (Figures 2(c) and 2(d)). To increase model stability, multivariate Cox analysis was performed by stepwise method. Finally, six IRGs were selected to form the IRG risk model (Figure 2(e)); three IRGs (*SSP1*, *ADAMTS5*, and *EPO*), with HRs > 1, were associated with increased risk, while the other three IRGs (*CXCR3*, *TNFRSF13C*, and *CRYAA*) were protective genes with HRs < 1 in TCGA cohort. The risk score was estimated as follows: risk score = (0.11026×expression level of SPP1) + (-0.18773×expression level of CXCR3) + (0.30501×expression level of ADAMTS5) + (0.12363×expression level of EPO) + (-0.29483×expression level of TNFRSF13C) + (-0.38391×expression level of CRYAA).

The C-index for TCGA and ICGC cohorts was 0.753 and 0.680, respectively. In TCGA cohort, the risk score's optimal cut-off value (0.230) was determined by the *survminer* R package (Figure 2(f)). Based on the cut-off value, 335 tumor samples were divided into the high-risk and the low-risk groups. In the same way, 231 tumor samples from the ICGC cohort were also divided into two risk groups, and the cut-off value was -5.10.

**Figure 3:**
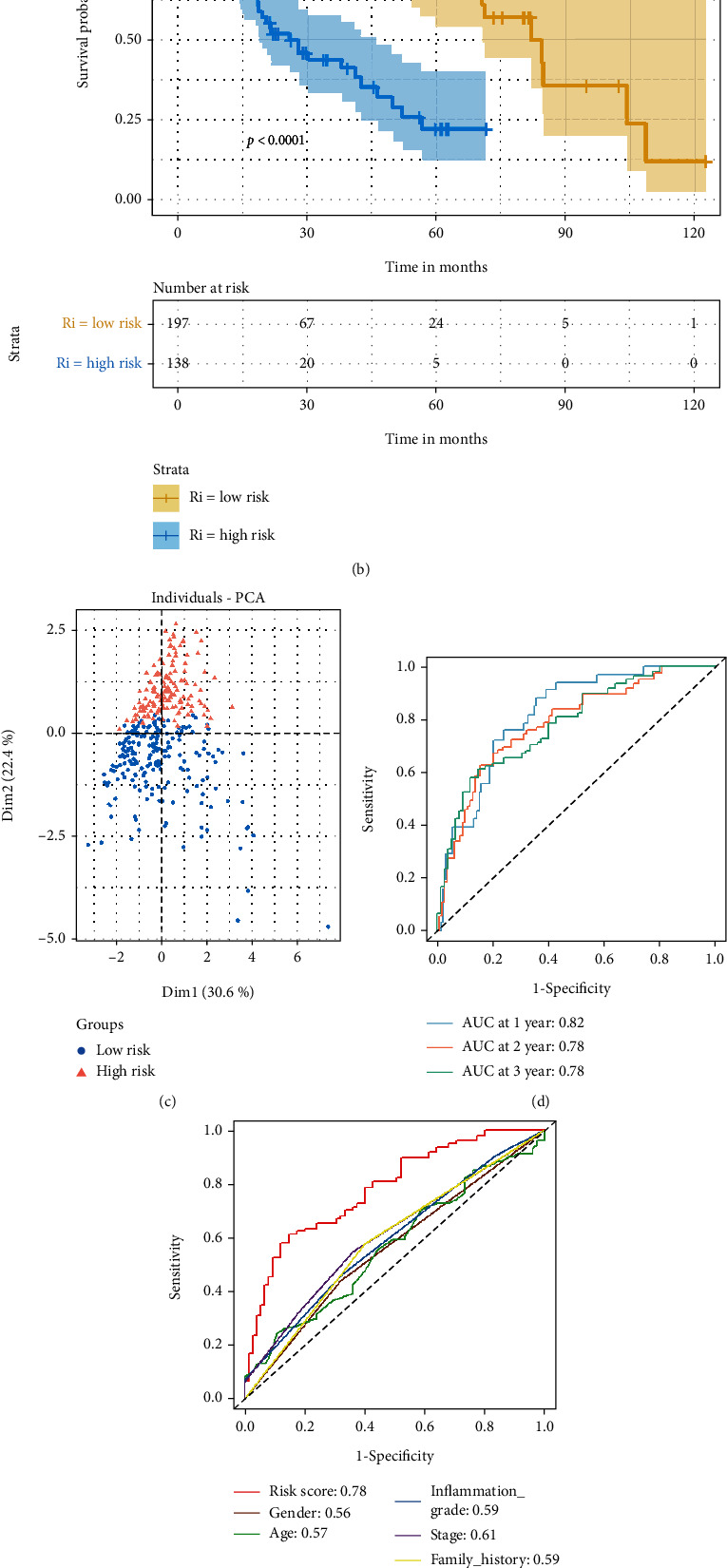
Performance assessment of the inflammation-related genes risk model in TCGA cohort. (a) Distribution of patients based on the risk score and the survival status (low-risk group: on the left side of the dotted line; high-risk group: on the right side of the dotted line). (b) The Kaplan-Meier curves for the OS. (c) The PCA plot for the HCC patients based on the six inflammation-related genes used to construct the risk model. (d) The ROC curve was used to display the predictive efficiency of the risk score for patient survival. (e) The ROC curve was used to display the predictive efficiency of the risk score and other clinical information for patient 3-year survival. (f) The univariate Cox analysis of the risk score and other clinical information in TCGA cohort. (g) The multivariate Cox analysis of the risk score and other clinical information in TCGA cohort.

**Figure 4:**
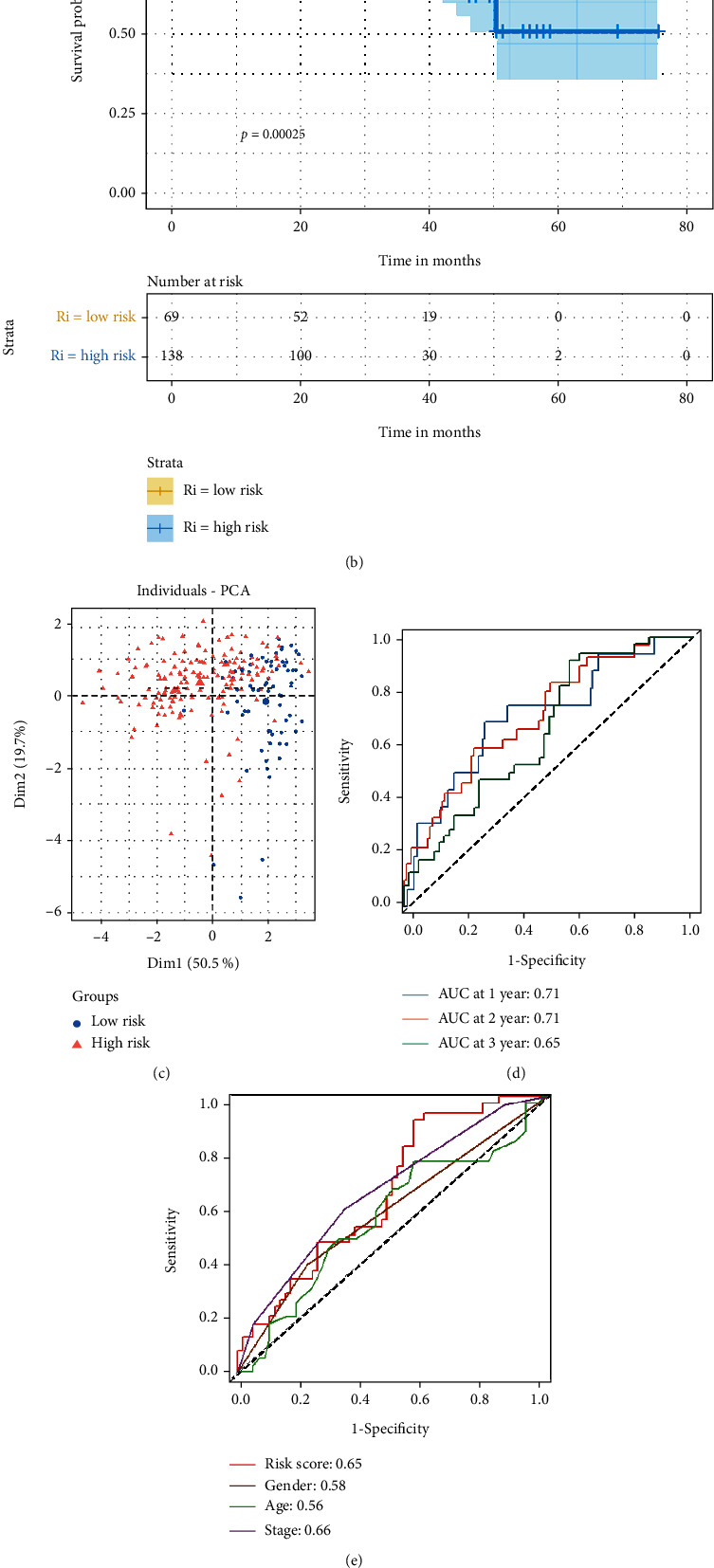
Validation of the inflammation-related genes risk model in the ICGC cohort. (a) Distribution of patients based on the risk score and the survival status (low-risk population: on the left side of the dotted line; high-risk population: on the right side of the dotted line). (b) The Kaplan-Meier curves for the OS. (c) The PCA plot for the HCC patients based on the six inflammation-related genes used to construct the risk model. (d) The ROC curve was used to display the predictive efficiency of the risk score for patient survival. (e) The ROC curve displayed the predictive efficiency of risk score and other clinical information for patient 3-year survival. (f) The univariate Cox analysis of the risk score and other clinical information for the ICGC cohort. (g) The multivariate Cox analysis of the risk score and other clinical information for the ICGC cohort.

In TCGA and ICGC cohorts, the proportion of dead patients was higher, and the survival time was shorter in the high-risk group than in the low-risk group (Figures 3(a) and 4(a)). The OS of patients was significantly lower in the high-risk group than in the low-risk group (*P* < 0.05) (Figures 3(b) and 4(b)). PCA based on the six IRGs showed that patients in two risk groups were distributed in different regions (Figures 3(c) and 4(c)).

Next, time-dependent ROC analysis was applied to evaluate the sensitivity and specificity of the risk model. The area under the ROC (AUC) of the risk score was 0.82 for 1-year, 0.78 for 2-year, and 0.78 for 3-year survival in TCGA cohort (Figure 3(d)). In the ICGC cohort, the AUC was 0.71 for 1-year, 0.71 for 2-year, and 0.65 for 3-year survival (Figure 4(d)). Besides, in TCGA cohort, the AUC of the risk score for 3-year survival was larger than that of other clinical features (the AUC of other clinical features for 3-year survival was less than 0.60) (Figure 3(e)). In the ICGC cohort, the AUC of the risk score for 3-year survival was similar to the AUC of tumor stage (the risk score: 0.65, tumor stage: 0.66), and higher than that of other clinical features (the AUC of other clinical features for 3-year survival were less than 0.60) (Figure 4(e)).

Then, we extracted patients' clinical information from TCGA cohort (age, gender, family history, inflammation grade, and tumor stage) and the ICGC cohort (age, gender, and tumor stage). In order to learn the independent prognostic value of the risk score, clinical information was analyzed in combination with the risk score by univariate and multivariate Cox regression analyses. In univariate Cox analysis, the risk scores of both cohorts were significantly correlated with OS (TCGA cohort: HR = 2.718, 95%CI = 2.064 − 3.58, *P* < 0.001; ICGC cohort: HR = 1.159, 95%CI = 1.080 − 1.242, *P* < 0.001) (Figures 3(f) and 4(f)). After adjustment for confounding factors, the multivariate Cox analysis showed that the risk score was an independent prognostic factor for OS (TCGA cohort: HR = 2.660, 95%CI = 1.850 − 3.830, *P* < 0.001; ICGC cohort: HR = 1.180, 95%CI = 1.040 − 1.340, *P* = 0.012) (Figures 3(g) and 4(g)).

### 3.3. Correlation of the Risk Score with Clinicopathologic Features

**Figure 5:**
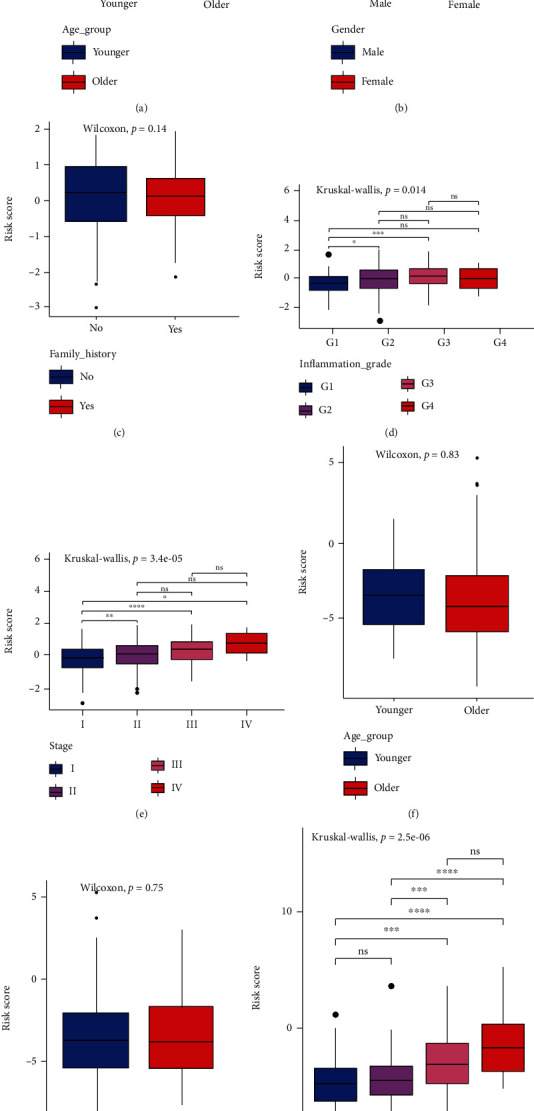
Correlation of the risk score with clinicopathologic features. (a–e) The differences in risk scores between different age groups, genders, family histories, inflammation grades, and tumor stages in TCGA cohort. (f–h) The differences in risk scores between different age groups, genders, and tumor stages in the ICGC cohort. ^∗^*P* < 0.05, ^∗∗^*P* < 0.01, ^∗∗∗^*P* < 0.001, ns: *P* > 0.05.

The boxplot was used to display the relationship of the risk score with the different clinical features. Patients aged < 62 years (median age of the patients in TCGA cohort) were defined as younger patients. The differences of the risk score in different age groups, genders, and family histories were not statistically significant in TCGA cohort (all *P* > 0.05, Figures 5(a)–5(c)). However, the risk scores were significantly correlated with inflammation grades and tumor stages and elevated with increasing inflammation grades and tumor stages in TCGA cohort (all *P* < 0.05) (Figures 5(d) and 5(e)); the same results were obtained in the ICGC cohort (*P* < 0.05) (Figures 5(f)–5(h)).

**Figure 6:**
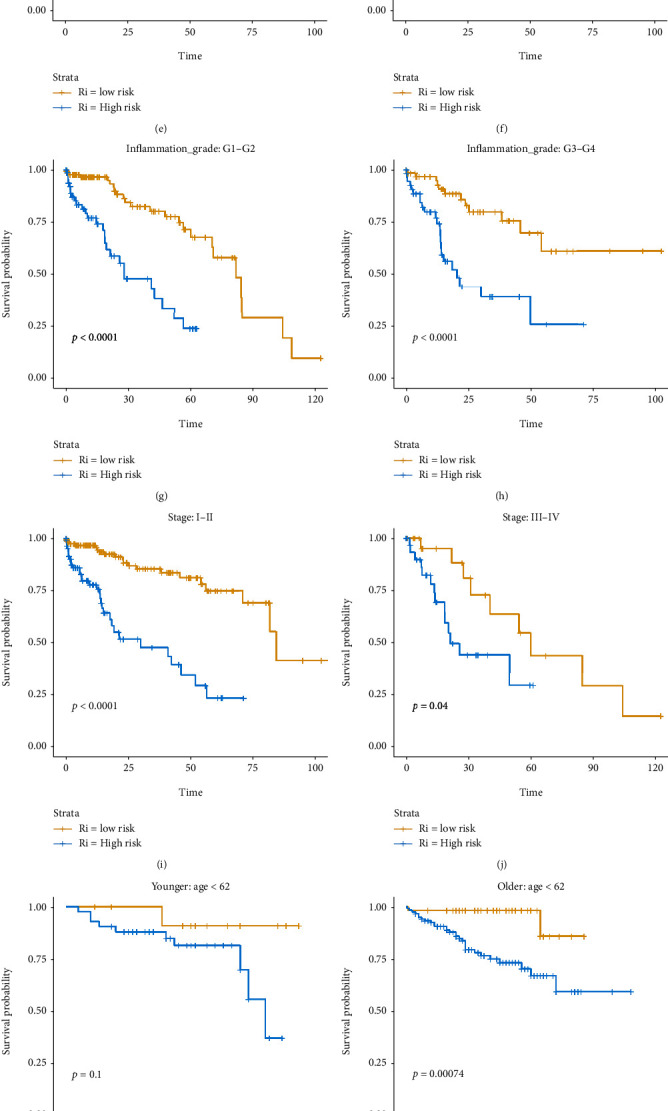
Survival analysis for different clinical features in the two risk groups. (a–j) Survival analysis for different clinical features in the two risk groups in TCGA cohort. (k–p) Survival analysis for different clinical features of the two risk groups in the ICGC cohort.

To explore the relationship of the risk score with the OS in patients with different clinical features, we conducted the Kaplan-Meier analysis and log-rank test. The results showed that the OS of the patients with different ages (Figures 6(a) and 6(b)), genders (Figures 6(c) and 6(d)), family histories (Figures 6(e) and 6(f)), inflammation grades (Figures 6(g) and 6(h)), and tumor stages (Figures 6(i) and 6(j)) was negatively correlated with the risk score. The OS of the patients was lower in the high-risk group than in the low-risk group of TCGA cohort (*P* < 0.05). In the ICGC cohort, the relationship of the risk score with the OS of patients with different ages, genders, and tumor stages was similar to those in TCGA cohort. Although the *P* values in the younger group and the stages III-IV were above 0.05, this may be due to the small sample size (Figures 6(k)–6(p)). Overall, these findings suggested that the IRG risk model was stable and could be used to predict the OS of HCC patients with different clinical characteristics.

### 3.4. Functional Enrichment Analysis of the DEGs between Two Risk Groups in TCGA Cohort

**Figure 7:**
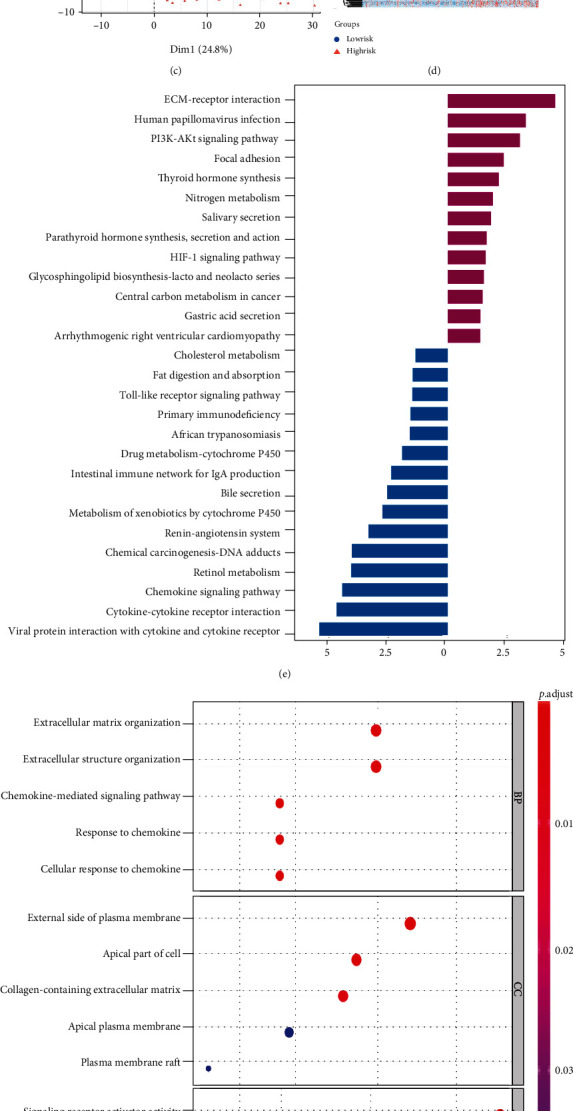
Identification and function enrichment analysis of the DEGs between two risk groups in TCGA cohort. (a) The overlapping upregulated DEGs were screened by the *DESeq2*, *edgeR*, and *limma* R packages. (b) The overlapping downregulated DEGs were screened by the DESeq2, edgeR, limma R packages. (c) The PCA plot for the HCC patients based on the DEGs. (d) The heatmap showed the expression levels of the DEGs; the redder indicates higher gene expression; the bluer indicates lower gene expression. The blue section of the annotation bar represents the low-risk group, and the red section the high-risk group. (e) The barplot graph for KEGG pathways. The left bar means the pathways associated with the downregulated DEGs, and the right bar represents the pathways related to the upregulated DEGs. The longer bar means the differences were more obvious. (f) The bubble graph for GO enrichment. The bigger bubble represents the more genes enriched, and the increasing depth of red means the differences were more obvious.

To further explore the differences in the gene functions and pathways between the two risk groups, we used the *DESeq2*, *edgeR*, and *limma* R packages to select the DEGs according to specific criteria (∣log2Fold − Change | >1 and *P* < 0.05). The results revealed that 167 DEGs were upregulated (Figure 7(a)), and 99 genes were downregulated (Figure 7(b)) in the high-risk group. PCA based on the DEGs showed patients in two risk groups were distributed in different regions (Figure 7(c)). The heatmap suggested that the expression levels of the DEGs were obviously different (*P* < 0.05, Figure 7(d)).

The KEGG pathway demonstrated that these DEGs were enriched in the inflammation, immune, and stromal-related signaling pathways, such as “primary immunodeficiency,” “chemokine signaling pathway,” “cytokine-cytokine receptor interaction,” and “ECM-receptor interaction” (Figure 7(e)). The enriched GO terms were divided into the biological process (BP), cell component (CC), and molecular function (MF) ontologies. The GO analysis revealed the DEGs were enriched in BPs, including “extracellular matrix organization,” “extracellular structure organization,” “chemokine-mediated signaling pathway,” “response to chemokine,” and “cellular response to chemokine.” For CC, DEGs were enriched in the “external side of plasma membrane”, “apical part of cell,” “collagen-containing extracellular matrix,” “apical plasma membrane,” and “plasma membrane raft.” In addition, MF analysis also displayed that the DEGs were enriched in “signaling receptor activator activity,” “receptor-ligand activity,” “chemokine receptor binding,” “CXCR chemokine receptor binding,” and “chemokine activity” (Figure 7(f)).

### 3.5. Comparison of the Immune Status between Two Risk Groups

**Figure 8:**
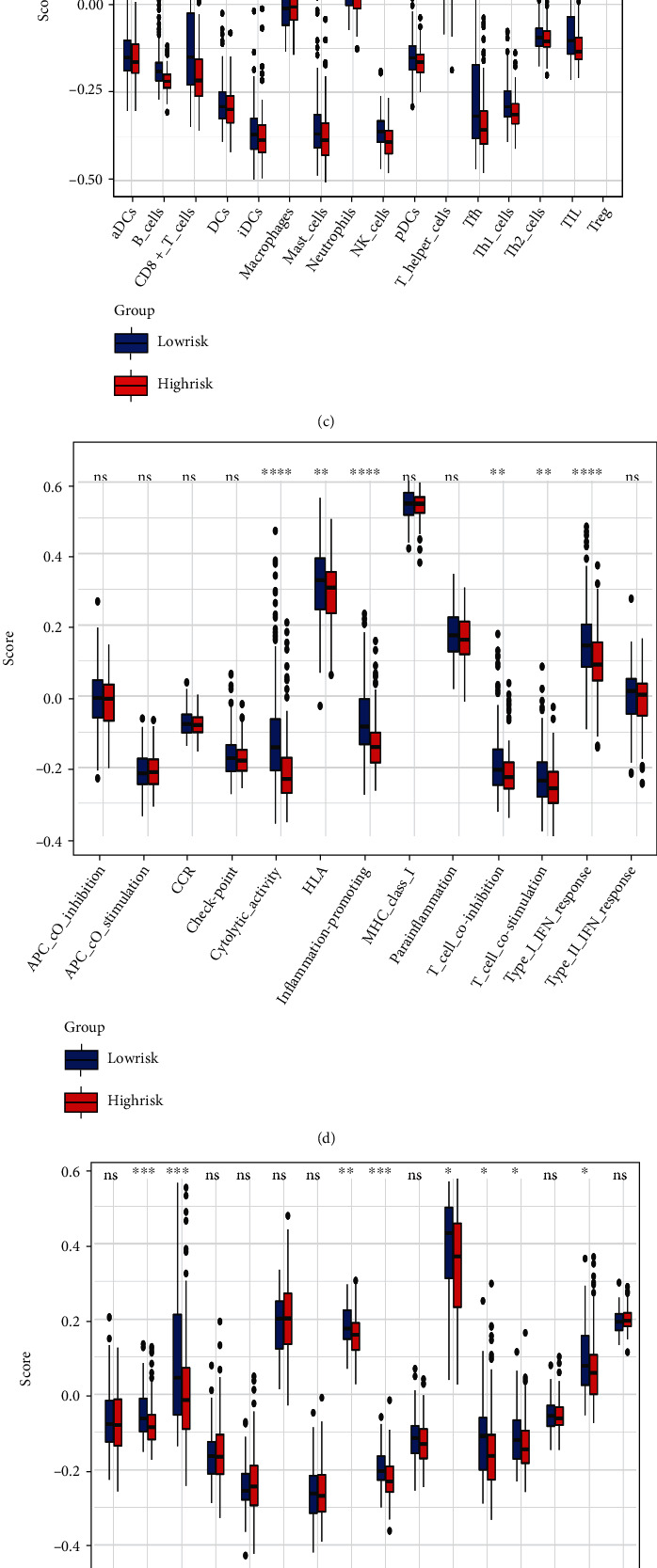
Correlation of the risk score and the immune cells. (a) Comparison of stromal scores, immune scores, and ESTIMATE scores between two risk groups in TCGA cohort. (b) Comparison of stromal scores, immune scores, and ESTIMATE scores between two risk groups in the ICGC cohort. (c) Comparison of ssGSEA scores of the immune cells between the two risk groups in TCGA cohort. (D) Comparison of ssGSEA scores of the immune pathways between the two risk groups in TCGA cohort. (e) Comparison of ssGSEA scores of the immune cells between the two risk groups in the ICGC cohort. (f) Comparison of ssGSEA scores of the immune pathways between the survival analysis of two groups of risk patients with different clinical characteristics and the two risk groups in the ICGC cohort. The blue box represents the low-risk group, and the red box represents the high- risk group, ^∗^*P* < 0.05, ^∗∗^*P* < 0.01, ^∗∗∗^*P* < 0.001, ns: *P* > 0.05.

We used the *ESTIMATE* algorithm to evaluate the immune, stromal, and ESTIMATE scores of TME [15]. In TCGA cohort, the results showed that the immune score was significantly lower in the high-risk group than in the low-risk group (*P* < 0.05). Yet, the differences in the stromal scores and ESTIMATE scores between the two risk groups were not statistically significant (*P* > 0.05, Figure 8(a)). In the ICGC cohort, the immune scores were also lower in the high-risk group, although the difference was not statistically significant (*P* > 0.05). Also, the stromal score and ESTIMATE score were comparable between the two risk groups (Figure 8(b)).

Next, we compared the immune cells and pathways between the two risk groups. In TCGA cohort, the high-risk group generally had significantly lower levels of infiltration of immune cells, including B cells, natural killer (NK) cells, CD8^+^ T cells, T helper cells, tumor-infiltrating lymphocytes (TIL), and neutrophils cells than the low-risk group (all *P* < 0.05, Figure 8(c)). Also, cytolytic activity, inflammation, the type I IFN response, T cell costimulation, T cell coinhibition, and HLA were lower in the high-risk group than in the low-risk group (*P* < 0.05) (Figure 8(d)). When assessing the scores of the immune cells and pathways between the two risk groups in the ICGC cohort, similar results were obtained (Figures 8(e) and 8(f)).

### 3.6. Assessment of the Predictive Value of the Risk Score for the Efficacy of Immunotherapy

**Figure 9:**
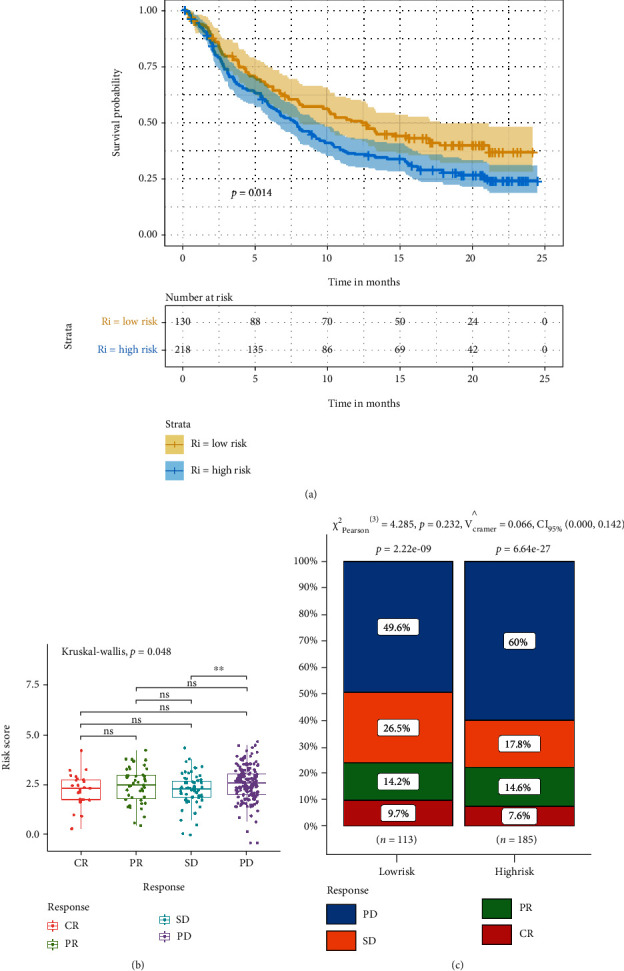
The role of the risk score in immunotherapeutic responses. (a) Survival analysis for two risk groups (*P* = 0.014, log-rank test) in the IMvigor210 cohort. (b) The difference of the risk score in different anti-PD-L1 clinical response groups. (c) The proportion of patients with response to PD-L1 blockade therapy in two risk groups. CR: complete response, PR: partial response, SD: stable disease, PD: progressive disease. ^∗^*P* < 0.05, ^∗∗^*P* < 0.01, ^∗∗∗^*P* < 0.001, ns: *P* > 0.05.

The above results showed that immune cells levels and immune signaling pathways were lower in high-risk groups than in low-risk groups. These findings suggested that the risk score might predict the effect of immunotherapy. Therefore, we used the IMvirgor210 cohort to assess the predictive value of the risk score for the efficacy of immunotherapy [17]. The *survminer* R package also determined the cut-off value (2.292). The low-risk group showed a significant clinical benefit and obviously prolonged survival (Figure 9(a)). (*P* < 0.05) Patients with partial response (PR) exhibited a higher risk than patients with complete response (CR), although the difference was not significant (*P* > 0.05). Yet, patients with progressive disease (PD) exhibited a higher risk than patients with stable disease (SD) (*P* < 0.05, Figure 9(b)). We also observed that the percentage of the patients with PD was higher in the high-risk group than in the low-risk group (60% vs. 49.6%), although the difference was not statistically significant (*P* > 0.05, Figure 9(c)). These findings suggested that the risk score could be used to assess the efficacy of immunotherapy.

## 4. Discussion

Inflammation is closely related to liver cancer, especially chronic hepatitis, which is one of the crucial factors in liver cancer formation. Chronic inflammation damages liver epithelial cells, leading to substantial cell proliferation [18]. At the same time, inflammation induces reactive oxygen species (ROS) and the damage of deoxyribonucleic acid (DNA), which increases the frequency of DNA mutations. When the rate of cell proliferation increases, along with DNA mutations, the incidence of malignant transformation increases accordingly [18]. However, so far, only a few studies have reported on the characteristics of IRGs in liver cancer tissues. In the present study, we constructed a new IRG risk model to predict HCC prognosis.

The IRG risk model was constructed using 6 IRGs, including secreted phosphoprotein 1(*SSP1*), C-X-C motif chemokine receptor 3 (*CXCR3*), ADAM metallopeptidase with thrombospondin type 1 motif 5 (*ADAMTS5*), erythropoietin (*EPO*), TNF receptor superfamily member 13C (*TNFRSF13C*), and crystallin alpha A (*CRYAA*).

*SPP1* is expressed in most human tissues, including the brain, vascular tissue, kidney, and liver [19, 20], and has been reported to regulate cancer cell proliferation through the activation of the MAPK pathway. It can also mediate HCC metastasis by inducing MMP-2 production/activation and NF-*κ*B translocation [21, 22].

*EPO* is a hypoxic-reactive cytokine [23]. Hypoxia is a common feature of TME, which increases the activity of a hypoxia-inducible factor (*HIF*) binding to a cis-acting DNA hypoxia response element (*HRE*) to activate *EPO* transcription [23, 24]. *EPO* can involve in the development of tumors by promoting tumor angiogenesis and the growth of tumor cells [25].

The exact role of *ADAMTS5* in HCC remains unclear. Li et al. [25] have reported that low expression of *ADAMTS5* protein is associated with HCC progression and poor prognosis. However, multiple online sites show high *ADAMTS5* expression as an independent risk factor for the development of HCC [26]. Furthermore, Zhu et al. [26] confirmed that *ADAMTS5* might promote tumor metastasis through biological processes affecting the extracellular matrix (ECM). These data are consistent with our findings.

*CRYAA*, also known as *HspB4*, is a well-known antiapoptotic protein [27, 28]. Studies have shown that its role in tumors depends on the type of tumor [29–32]. Some studies have suggested that *HSPB4* can promote retinoblastoma and sebaceous adenocarcinoma progression through antiapoptotic effects [33, 34]. In contrast, *HspB4* is expressed at a moderate level in the normal pancreas and significantly downregulated in pancreatic cancer [30]. It can delay the progression of the tumor by regulating the activity of ERK MAP kinase [35]. However, further experiments are needed to verify the role of HSP4 in the formation and development of HCC.

*CXCR3* is a chemokine receptor that is mainly expressed on CD4^+^ and CD8^+^ T cells and partly expressed on other cells, including epithelial cells [36, 37]. In the CD4^+^ subpopulation, *CXCR3* is most abundant in proinflammatory Th1 cells [38–41]. Thus, the expression level of *CXCR3* is related to the abundance of immune cell infiltration in TME [42].

*TNFRSF13C*, also known as B-cell activating factor receptor (BAFF-R), is expressed almost exclusively on B cells [43]. It is a key receptor involved in B cells' successful survival and maturation, which determines that its expression levels are closely correlated with the abundance of B cells in TME [44]. A recent study has found a significantly lower percentage of B cells expressing BAFF-R in HBV-associated HCC patients than non-HCC patients, suggesting an important role for BAFF-R in developing HCC in HBV-infected patients [45].

In summary, *CXCR3* and *TNFRSF13C* were associated with immune cell abundance in TME, while *ADAMTS5* was closely related to the change of stromal components in TME. *SPP1* and *EPO* promote tumor progression in a variety of ways. The mechanism of *CRYAA* in liver cancer has not been reported, which also provides a new direction for further understanding of the development of HCC.

The risk score calculated by the IRG risk model in our study was identified as an independent risk factor for HCC prognosis. The higher the risk score, the worse the prognosis and the lower the OS. Lin et al. [14] also reported an IRG risk model with eight IRGs used for prognostic prediction (the risk score =0.118×expression level of SLC7A1+0.114×expression level of RIPK2+0.113×expression level of NOD2+0.022×expression level of ADORA2B+0.058×expression level of MEP1A+0.051×expression level of ITGA5+0.016×expression level of P2RX4+0.018×expression level of SERPINE1). However, the risk model in Lin et al.'s study was constructed based on 8 IRGs, compared to 6 IRGs used in this study. In addition, compared to the new risk model built in this study, the AUC of the risk model built by Lin et al. was relatively low. The AUC of Lin et al.'s risk score was only 0.685 for 1-year, 0.626 for 2-year, and 0.605 for 3-year survival in TCGA cohort. Moreover, in the ICGC cohort, the AUC of Lin's risk score was 0.649 for 1-year, 0.649 for 2-year, and 0.681 for 3-year survival. In the present study, the AUCs of a risk score for 1-year survival, 2-year survival, and 3-year survival were above 0.750 in TCGA cohort; in the ICGC cohort, the AUCs of the risk score for 1-year survival and 2-year survival were above 0.700, and the AUC of the risk score for 3-year survival was 0.650, which is similar to the risk score in Lin et al.'s study. These findings indicated that the risk model in our study had a higher predicting value for HCC than the risk model presented by Lin et al.

Besides, the results of KEGG and GO enrichment analysis based on the DEGs between two risk groups revealed the DEGs were associated with inflammation, immune, and stromal-related pathways. These findings suggested that TME, including immune and stromal, was different in the high-risk group compared to the low-risk group. TME is defined as the cellular and physical environment surrounding the primary tumor, which has cellular components like inflammatory and immune cells, stromal components like ECM proteins, soluble cellular factors, etc. [46, 47] In TME, tumor purity is a concept closely related to the immune cells and stromal cells, which refers to the proportion of the tumor cells in the tumor tissue [48]. Yoshihara et al. [15] used an ESTIMATE method to deduce the tumor purity. ESTIMATE score is the combination of stromal score and immune score, the primary basis for predicting tumor purity [15]; the lower ESTIMATE score, the higher tumor purity [48]. In the present study, the tumor purity was similar between the two risk groups, but the immune score was lower in the high-risk group than in the low-risk group. And most of the immune cells and immune-related pathways were reduced in the high-risk group, suggesting low immune levels. The same results were obtained in the ICGC cohort. It is well known that some immune cells have important roles in antitumor immunity, such as CD8^+^ T cells [49], NK cells [50], and B cells [51]. The decrease in immune response was closely related to the poor prognosis of HCC, which may also be one reason for the poor prognosis of patients in the high-risk group [52].

Since the risk score was closely associated with the low immune infiltrating, we sought to further explore whether the risk model could be used to assess the immunotherapy efficacy in tumor patients [17]. We used the risk score to analyze the tumor patient in the IMvigor210 cohort. The risk score was closely associated with patient prognosis, and in the high-risk group, 60% of patients suffered disease progression. These results suggested that risk scores can partly predict the efficacy of tumor immunotherapy. The higher the risk score, the worse the efficacy of immunotherapy. Therefore, it is necessary to actively monitor the patients with high-risk scores and combine multiple treatments to achieve a better antitumor effect.

Our study has significant clinical application values. The inflammation-related risk score could be used as an independent risk factor for predicting the outcomes of HCC patients. This model can be applied to identify tumors with low immune levels and indicate the efficacy of immunotherapy. Strengthening the study of these six IRGs may advance the understanding of tumorigenesis.

However, this study also has several limitations. First, this risk model was not confirmed by the prospective experimental data. Second, we failed to validate the predictive value of the risk model for the immunotherapy efficacy in an HCC-related immunotherapy cohort due to the fewer data on the HCC-related immunotherapy cohort.

## 5. Conclusions

The IRG risk model consisting of 6 IRGs is closely related to the prognosis of HCC, which could predict the HCC prognosis more accurately than Lin et al.'s risk model. Therefore, the evaluation of patients with the risk model was a more practical approach that furthered our understanding of the status of immune infiltration in tumor tissue. This approach can also help us to better evaluate the efficacy and to guide immunotherapy. In our future study, we plan to further validate the expression of 6 IRGs in tumor tissues and validate the clinical application value of this risk model in a prospective cohort.

## Figures and Tables

**Table 1:** The clinical information of the HCC patients.

	TCGA		ICGA		Total	
	Alive	Death	Alive	Death	Alive	Death
	(*N* = 246)	(*N* = 89)	(*N* = 189)	(*N* = 42)	(*N* = 435)	(*N* = 131)
Sex						
Male	171 (69.5%)	51 (57.3%)	144 (76.2%)	26 (61.9%)	315 (72.4%)	77 (58.8%)
Female	75 (30.5%)	38 (42.7%)	45 (23.8%)	16 (38.1%)	120 (27.6%)	54 (41.2%)
Age (years)						
Mean (SD)	59.5 (12.8)	64.5 (13.3)	67.3 (10.3)	67.2 (9.55)	62.9 (12.4)	65.4 (12.3)
Median [min, max]	61.0 [17.0, 85.0]	66.0 [24.0, 88.0]	69.0 [31.0, 89.0]	68.5 [37.0, 83.0]	65.0 [17.0, 89.0]	67.0 [24.0, 88.0]
Missing	1 (0.4%)	1 (1.1%)	0 (0%)	0 (0%)	1 (0.2%)	1 (0.8%)
Age group						
Younger	132 (53.7%)	33 (37.1%)	44 (23.3%)	11 (26.2%)	176 (40.5%)	44 (33.6%)
Older	113 (45.9%)	55 (61.8%)	145 (76.7%)	31 (73.8%)	258 (59.3%)	86 (65.6%)
Missing	1 (0.4%)	1 (1.1%)	0 (0%)	0 (0%)	1 (0.2%)	1 (0.8%)
Stage						
I	128 (52.0%)	38 (42.7%)	35 (18.5%)	1 (2.4%)	163 (37.5%)	39 (29.8%)
II	61 (24.8%)	16 (18.0%)	88 (46.6%)	17 (40.5%)	149 (34.3%)	33 (25.2%)
III	44 (17.9%)	20 (22.5%)	56 (29.6%)	15 (35.7%)	100 (23.0%)	35 (26.7%)
IV	1 (0.4%)	3 (3.4%)	10 (5.3%)	9 (21.4%)	11 (2.5%)	12 (9.2%)
Missing	12 (4.9%)	12 (13.5%)	0 (0%)	0 (0%)	12 (2.8%)	12 (9.2%)
Family history						
No	143 (58.1%)	37 (41.6%)	NA	NA	143 (32.9%)	37 (28.2%)
Yes	66 (26.8%)	45 (50.6%)	NA	NA	66 (15.2%)	45 (34.4%)
Missing	37 (15.0%)	7 (7.9%)	NA	NA	226 (52.0%)	49 (37.4%)
Inflammation grade						
G1	36 (14.6%)	11 (12.4%)	NA	NA	36 (8.3%)	11 (8.4%)
G2	119 (48.4%)	40 (44.9%)	NA	NA	119 (27.4%)	40 (30.5%)
G3	82 (33.3%)	30 (33.7%)	NA	NA	82 (18.9%)	30 (22.9%)
G4	7 (2.8%)	5 (5.6%)	NA	NA	7 (1.6%)	5 (3.8%)
Missing	2 (0.8%)	3 (3.4%)	NA	NA	191 (43.9%)	45 (34.4%)
Time (months)						
Mean (SD)	21.1 (23.0)	24.4 (24.5)	29.1 (13.1)	17.8 (14.0)	24.6 (19.7)	22.3 (21.8)
Median [min, max]	12.3 [0.100, 123]	18.5 [0.300, 109]	29.0 [3.00, 72.0]	16.0 [0.333, 48.0]	20.0 [0.100, 123]	17.0 [0.300, 109]
Risk score						
Mean (SD)	-0.141 (0.828)	0.389 (0.843)	-3.86 (2.28)	-2.44 (2.55)	-1.76 (2.46)	-0.517 (2.07)
Median [min, max]	-0.0897 [-2.99, 1.74]	0.320 [-2.34, 1.95]	-4.03 [-8.86, 5.30]	-2.83 [-7.04, 3.73]	-0.884 [-8.86, 5.30]	0.0940 [-7.04, 3.73]
Risk group						
Low risk	160 (65.0%)	37 (41.6%)	66 (34.9%)	3 (7.1%)	226 (52.0%)	40 (30.5%)
High risk	86 (35.0%)	52 (58.4%)	123 (65.1%)	39 (92.9%)	209 (48.0%)	91 (69.5%)

**Table 2:** The clinical information of the patient in the IMvirgor210 cohort.

Characteristics	Alive	Death	Total
	(*N* = 116)	(*N* = 232)	(*N* = 348)
Sex			
Male	95 (81.9%)	177 (76.3%)	272 (78.2%)
Female	21 (18.1%)	55 (23.7%)	76 (21.8%)
Response			
CR	24 (20.7%)	1 (0.4%)	25 (7.2%)
PR	39 (33.6%)	4 (1.7%)	43 (12.4%)
SD	23 (19.8%)	40 (17.2%)	63 (18.1%)
PD	23 (19.8%)	144 (62.1%)	167 (48.0%)
Missing	7 (6.0%)	43 (18.5%)	50 (14.4%)
Time			
Mean (SD)	18.0 (5.82)	6.35 (5.05)	10.2 (7.66)
Median [min, max]	20.1 [0.197, 24.5]	5.03 [0.230, 21.2]	8.05 [0.197, 24.5]
Risk score			
Mean (SD)	2.31 (0.814)	2.58 (0.816)	2.49 (0.824)
Median [min, max]	2.36 [0.0690, 4.25]	2.63 [-0.331, 4.67]	2.47 [-0.331, 4.67]
Risk group			
Low risk	55 (47.4%)	75 (32.3%)	130 (37.4%)
High risk	61 (52.6%)	157 (67.7%)	218 (62.6%)

Note: CR: complete response, PR: partial response, SD: stable disease, PD: progressive disease.

## Data Availability

The data could be downloaded at https://portal.gdc.cancer.gov/ and https://dcc.icgc.org/projects/LIRI-JP, and the code used in this study is available from the corresponding author on reasonable request.
